# Monkeypox: A Growing Concern for Pulmonary Health

**DOI:** 10.7759/cureus.68614

**Published:** 2024-09-04

**Authors:** Vasiliki E Georgakopoulou, Konstantinos Dodos, Kyriakos Tarantinos

**Affiliations:** 1 Department of Pathophysiology/Pulmonology, Laiko General Hospital, Athens, GRC; 2 Department of Respiratory Medicine, General Oncological Hospital of Kifissia “Agioi Anargyroi’’, Athens, GRC; 3 First Department of Respiratory Medicine, Sismanogleio Hospital, Athens, GRC

**Keywords:** public health, ards, respiratory complications, pulmonary health, monkeypox

## Abstract

The re-emergence of monkeypox as a significant global health concern has highlighted the need to reassess its clinical presentation, particularly its impact on pulmonary health. Traditionally associated with dermatological symptoms, recent outbreaks in non-endemic regions have underscored the virus's potential for widespread transmission and respiratory complications. Emerging evidence indicates that monkeypox infection can lead to respiratory symptoms such as cough, sore throat, and, in severe cases, pneumonia and acute respiratory distress syndrome (ARDS).

The virus's capacity for respiratory transmission, coupled with the potential for coinfection with other respiratory pathogens, poses significant challenges for clinical management. Understanding the pathophysiology of monkeypox's impact on the lungs, which may involve direct viral invasion and immune-mediated damage, is critical for developing effective public health strategies. As monkeypox continues to spread, recognizing and addressing its respiratory complications will be essential in mitigating severe outcomes, particularly in vulnerable populations with pre-existing pulmonary conditions.

## Editorial

The re-emergence of monkeypox as a significant public health threat has raised alarms across the globe. Originally confined to endemic regions in Central and West Africa, recent outbreaks in non-endemic countries have underscored the virus's capacity for widespread transmission. While monkeypox has been traditionally associated with dermatological manifestations, emerging evidence suggests that its implications for pulmonary health deserve closer attention [1].

Monkeypox is caused by the monkeypox virus, an Orthopoxvirus closely related to the variola virus, which causes smallpox. Human-to-human transmission of the monkeypox virus can occur through direct contact with infectious skin or mucocutaneous lesions, as well as through respiratory droplets during prolonged face-to-face contact. Additionally, the virus can spread via fomites, such as contaminated bedding or clothing. It is also possible for transmission to occur through contact with bodily fluids from an infected person. Though primarily transmitted through close contact with infected individuals or animals, the virus's potential for respiratory transmission is a growing concern. Respiratory droplets can carry the virus, leading to its spread in close quarters, particularly in households and healthcare settings. This route of transmission places individuals at risk of respiratory complications, a topic that has been less explored in the literature but is gaining attention with the rising number of cases.

Monkeypox is primarily diagnosed through clinical evaluation and confirmed via laboratory testing. The diagnosis is usually established by detecting the virus's DNA through polymerase chain reaction (PCR) testing of samples from skin lesions, such as swabs from vesicles, pustules, or crusts. Serological tests can also be used to detect antibodies, although cross-reactivity with other orthopoxviruses may complicate interpretation. Accurate and timely diagnosis is crucial for effective patient management and public health response [2]. Recent studies have indicated that monkeypox infection can lead to respiratory symptoms such as cough, sore throat, and, in severe cases, pneumonia. These symptoms are particularly concerning in vulnerable populations, including those with pre-existing pulmonary conditions. A case report highlighted the development of acute respiratory distress syndrome (ARDS) in a patient with monkeypox, emphasizing the need for clinicians to be vigilant about respiratory complications in infected individuals [3].

The pathophysiology of monkeypox's impact on the lungs is not fully understood, but it is likely multifactorial. The virus can directly invade respiratory epithelial cells, leading to inflammation and damage. Additionally, the immune response to the virus, characterized by a robust release of cytokines, may contribute to lung injury. This cytokine storm, akin to that observed in severe coronavirus disease 2019 (COVID-19) cases, can exacerbate pulmonary inflammation and lead to complications such as ARDS [4]. Moreover, the potential for coinfection with other respiratory pathogens, including influenza and severe acute respiratory syndrome coronavirus 2 (SARS‑CoV‑2), cannot be overlooked. Such coinfections could exacerbate respiratory symptoms and complicate the clinical management of monkeypox. Therefore, it is crucial to consider comprehensive diagnostic evaluations in patients presenting with respiratory symptoms and monkeypox, particularly in regions experiencing concurrent outbreaks of other respiratory diseases [5].

Despite these concerning developments, significant gaps remain in our understanding of the respiratory implications of monkeypox. Research is urgently needed to elucidate the mechanisms by which monkeypox affects the respiratory system, particularly in distinguishing direct viral effects from immune-mediated damage. Studies should also explore the potential for long-term pulmonary sequelae in survivors of severe monkeypox, as well as the effectiveness of current antiviral treatments in mitigating respiratory complications. Additionally, there is a need for robust epidemiological studies to assess the true burden of respiratory symptoms in monkeypox cases, especially in diverse population groups and settings. Identifying risk factors for severe respiratory involvement, such as genetic predispositions or environmental factors, could help guide targeted interventions. Furthermore, the development of animal models that accurately reflect human respiratory disease in monkeypox could be instrumental in testing potential therapies and preventive measures.

The global spread of monkeypox necessitates a re-evaluation of its clinical presentation and the potential for severe respiratory complications. Public health strategies should include guidelines for the identification and management of respiratory symptoms in monkeypox cases. Healthcare providers should be prepared to recognize the signs of pulmonary involvement and take appropriate measures to prevent severe outcomes, especially in patients with underlying lung conditions.

In conclusion, while monkeypox has historically been viewed through the lens of dermatological symptoms, its potential impact on pulmonary health should not be underestimated. As the virus continues to spread, understanding and mitigating its respiratory complications will be crucial in managing this evolving public health threat.
